# Three-wire technique for irreducible extension type Supracondylar humeral fracture in children

**DOI:** 10.1186/s13018-025-06543-z

**Published:** 2025-12-15

**Authors:** Mahmoud Badawy, Sami Ibrahim Sadek, Mohammad Hassan, Ahmed Mostafa Elnagar, Elsayed Shaheen, Ibrahim Abdellatif Algohiny

**Affiliations:** 1 Zagazig University, Menia El-Qamh, Sharkia 44111 Egypt; 2 Zagazig University, Zagazi, Sharkia 7510001 Egypt; 3 Zagazig University, El Husseiniya, Sharkia 44654 Egypt; 4 Alazhar University, Cairo, 3753450 Egypt

**Keywords:** Extension type supracondylar humeral fracture, Irreducible supracondylar humeral fracture, Closed reduction techniques for supracondylar humeral fracture

## Abstract

**Background:**

Irreducible extension type supracondylar humeral fracture [ESCHF] accounts for 3–15% of cases and often requires open reduction, which carries the risk of complications. This study describes and reports the outcomes of the three-wire technique for treating irreducible ESCHF.

**Methods:**

Twenty patients with irreducible ESCHF (8 girls and 12 boys) underwent closed reduction and percutaneous pinning using the three-wire technique. We inserted two K-wires in an unreduced position in the distal fragment and one proximal wire just above the olecranon fossa and used these three wires for manipulation and correction of sagittal, coronal, and rotational deformities at the fracture site.

**Results:**

All the fractures achieved acceptable closed reduction, with a mean operation time of 32.65 min. Radiological assessments showed favorable outcomes: the mean Baumann angle was 70.05 ± 1.70 degrees, the average carrying angle was 12.95 degrees, and the anterior humeral line dissected the capitellum at the middle third in 16 patients and at the anterior third in 4 patients. The complications included mild pin tract infections in five patients, all of which resolved within one week after K-wire removal.

**Conclusion:**

The three-wire technique is effective for managing irreducible ESCHF, providing good outcomes while avoiding the risks associated with open reduction.

## Introduction

Extension type supracondylar humeral fracture (ESCHF) accounts for approximately 97% of all supracondylar humeral fractures (SCHFs). Gartland classified these fractures into three types based on the degree of the displacement [2]. Leitch and coworkers [3] later added a type IV SCHF, which represents a multidirectional unstable fracture lacking a periosteal hinge.

Most displaced ESCHF patients are treated with closed reduction and percutaneous pinning. A minority of cases in which closed reduction fails require open reduction [4].

Swenson was the first to introduce the closed reduction technique for ESCHFs, and he depended on manipulation for anatomical correction of the deformity [5]. In 1955, Blount introduced the periosteal hinge concept; he relied on the intact medial and posterior periosteum for reduction. After satisfactory reduction, above elbow cast or back slab was then applied in 110–120^0^ flexion [6]. In 1974, Flynn combined Blount’s gentle closed reduction, and percutaneous pinning under an image intensifier, which is considered the gold standard method [7]. Gadgil et al. [8] used straight arm traction as a reduction method for supracondylar humeral fractures in children younger than 10 years of age, with 63% excellent results. Straight-arm longitudinal traction has fallen of favor by most surgeons due to prolonged hospital stays, increased costs and reported malreduction complications [9].

Irreducible ESCHF is defined as the inability to achieve or maintain an acceptable anatomical alignment after a maximum of two closed reduction trials. The criteria for acceptable reduction include restoration of the Baumann angle to within 5–10 degrees of the normal side, intact medial and lateral humeral columns in oblique views and the anterior humeral line passing through the middle third of the capitellum in the lateral view. Minimal rotational deformity is acceptable, with a difference of less than 2 mm in width between both fracture fragments at the fracture site in the lateral view [7, 10]. Inadequate fracture reduction or loss of reduction can result in cubitus varus deformity [11].

The rate of closed reduction failure reported in the literature is between 3% and 15%. This has been attributed to many factors, such as the severity of the injury, the fracture type, and soft tissue interposition [12–15].

Both repeated attempts at closed reduction and open reduction have their own complications. Repeated trials of closed reduction can increase the risk of compartment syndrome, neurovascular damage, and myositis ossificans [16]. Compared to closed reduction, open reduction is associated with worse functional outcomes, longer anesthesia time, scar formation, and poorer cosmetic results [14].

## Patient and methods

**Fig. 1:**
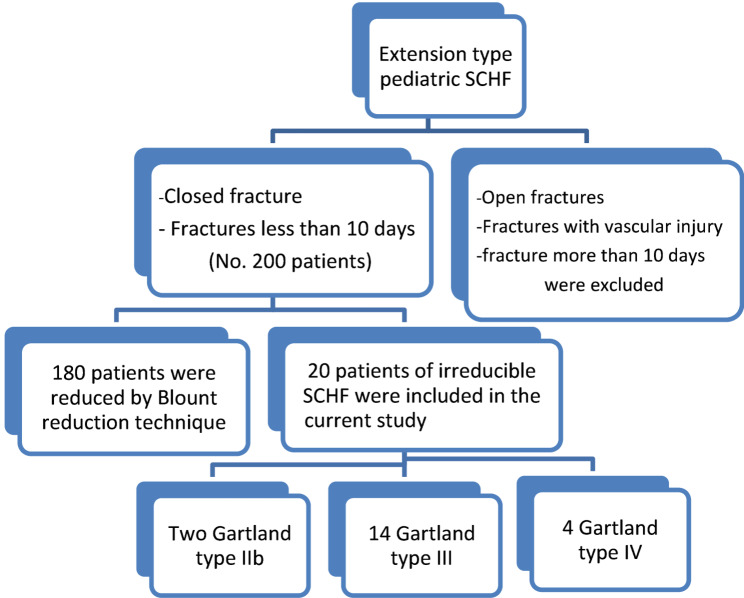
A consort flow diagram of the current study

This retrospective study was conducted on twenty irreducible ESCHF patients from 200 ESCHFs operated on between March 2021 and March 2024 at our institution. All patients were initially treated with an above elbow back slab, given adequate analgesia and underwent preoperative investigations. We included patients who had failed closed reduction of ESCHF after two trials of the gentle Blount reduction technique [6]. Patients with ESCHF more than 10 days after the time of injury were excluded because closed reduction is more difficult, and these patients are more susceptible to myositis ossificans. Additionally, open fractures and fractures associated with vascular injury were excluded, as open reduction is indicated in these cases. Informed consent was obtained from each child’s parent, and the study was approved by our Institutional Review Board (IRB) [ZU-IRB# 269/1-4-2024]. Figure 1 shows the consort flow diagram of the present study.

### Surgical technique

With the patient in a supine position and under general anesthesia, the affected upper extremity was sterilized and draped. One of the first, second, or last authors (pediatric orthopedic surgeons), with a single assistant, performed the initial closed reduction trials and the three-wire technique after failed reduction. The image intensifier tube was at the ipsilateral side of the fractured arm parallel to the operating table, and the TV was at the contralateral side. The surgeon was located in front of the injured arm, and the assistant was beside the patient’s ipsilateral shoulder with the image tube in between. The fluoroscopic checkpoints were for the position of the three wires at the prereduction phase, then to check the quality of reduction, and finally, to check the fixation stability.

**Fig. 2:**
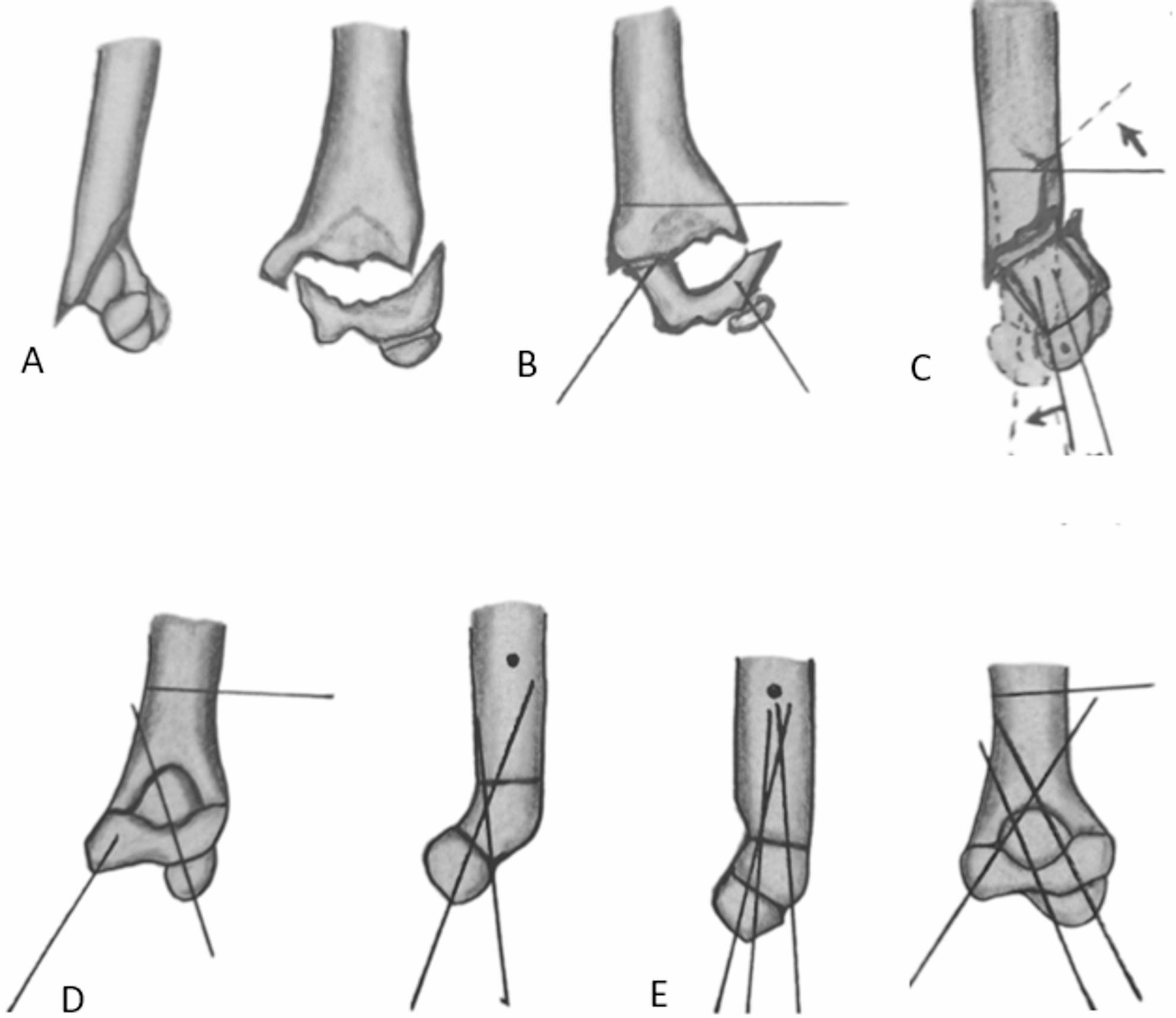
**A** irreducible ESCHF after 2 trials of closed reduction. **B** the three wires were inserted. **C** Correction of rotational and sagittal deformities via the three-wire technique. **D** The lateral K-wire was advanced to the proximal medial cortex. **E** the medial wire was then advanced, and a second lateral K-wire was inserted

**Fig. 3:**
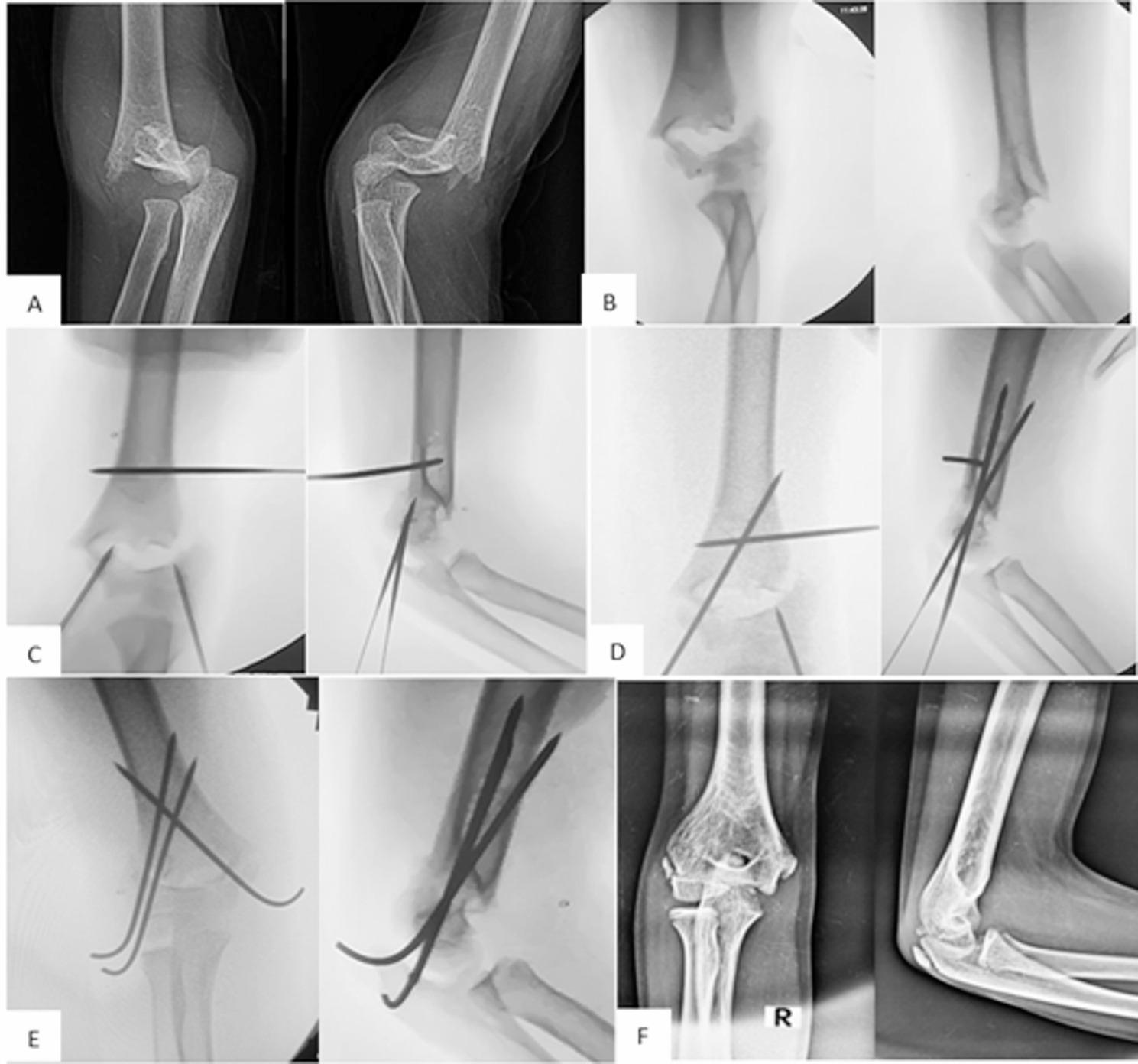
**A** A 13-year-old boy with displaced right-sided ESCHF. **B** After 2 trials of closed reduction with the traditional method. **C**–**E** Intraoperative images showing reduction of the fracture via the three-wire technique. **F** Follow-up plain X-rays with anterior‒posterior and lateral views showing fracture union in an acceptable position

Initially, two closed reduction trials were performed for all the cases. For those that remained irreducible, the three-wire technique was used (Fig. 2A). This technique followed the same principles described by the first author and coworkers for facilitating closed reduction of flexion-type SCHFs [17]. In the unreduced fracture position, two appropriately sized K-wires (1.2–1.8 mm), depending on the age of the patient and the size of his or her bone, were inserted into the distal fracture fragment: one in the lateral column and one in the medial column, both positioned short of the fracture line. The distal wires should be more or less central in the medullar canal in the lateral view and oriented approximately 30 to 55 degrees from the long axis of the humerus in the anteroposterior view. It should aim to engage a good grip in the proximal fracture fragment. The medial wire was inserted with elbow extension and anterior in the medial epicondyle to avoid injury to the ulnar nerve. A third K-wire was inserted into the proximal fracture fragment, approximately 1 cm above the olecranon fossa from a lateral to medial direction (Fig. 2B). To prevent injury to the radial nerve, the proximal wire was introduced posterior to the lateral edge of the humerus, as the radial nerve is located anteriorly between the brachioradialis and brachialis muscles in this region. The reduction sequence started with correction of the impaction, then the coronal plane, the sagittal plane, and finally the rotational plane deformities. The process began with traditional traction and countertraction, maintaining the elbow in 15–30 degrees of flexion. This was followed by correcting the coronal plane deformity and flexing the elbow while pushing the distal fragment anteriorly. The three-wire technique facilitated the correction of residual fracture deformities (Fig. 2C). Disimpaction of the fracture fragments was facilitated by pulling the distal wires distally and the proximal wire proximally. The technique effectively corrected the coronal and sagittal plane angulation but not the translation angle. The correction of the translation by wire manipulation was not effective. Additionally, using wires for correction of the translation would result in bending of the K-wires. Translation was corrected by both traction and manipulation of the distal fragment in the opposite direction of the translational deformity. The rotational deformity was corrected by rotating the proximal fragment internally or externally by the proximal joystick wire. The lateral view obtained by rotating the image intensifier itself. Rotation was evaluated intraoperatively and malrotation was considered if there was asymmetry in the width of the fracture ends more than 2 mm in the lateral view. The accuracy of the reduction was verified under the image intensifier. Once an acceptable reduction (good cortical alignment, no or minimal rotation, and the anterior humeral line passed at the middle third of the capitellum) was achieved, the lateral wire was advanced through the fracture site to engage the medial cortex proximally while maintaining reduction with the proximal and medial wires. The medial wire was subsequently advanced through the fracture site in a cross direction to the lateral wire to engage the lateral cortex proximally, again ensuring that the reduction was preserved with the aid of the proximal and lateral wires (Fig. 2D). If one of the distal wires was bent during manipulation, we replaced it with a new wire after fixation of the fracture with a nonbent wire. Additionally, we sometimes changed the position and trajectory of the k-wires if they did not engage a good grip in the proximal fragment. Finally, an additional lateral K-wire was introduced to secure the fixation of this unstable fracture (Fig. 2E). The stability of the reduction was confirmed by gently stressing the fracture under the image intensifier in all directions. Distal pulsation was checked, and a back slab was applied with the elbow in 90 degrees of flexion and the forearm in neutral rotation. Figure 3 illustrates the preoperative, intraoperative, and postoperative follow-up of a male patient with irreducible ESCHF.

### Postoperative care and follow-up

Clinically, we assessed the neurovascular status of the limb. The above-elbow back slab and the K-wires were maintained until full union (4–6 weeks post-operatively). After the K-wires extraction, passive and active elbow and wrist exercises were started until the full elbow and wrist range of motion and the full power of the flexors and extensors of the elbow were restored, which was usually obtained at the 3rd postoperative month.

Radiologically, plain radiographs in both anteroposterior and lateral views were obtained during the immediate postoperative period to assess the accuracy of the reduction. Follow-up radiographs were taken at the 4th and 6th weeks to assess fracture union, with additional imaging conducted at the final follow-up to evaluate the carrying angle, Baumann angle, and anterior humeral line.

### Data analysis

All data were collected, tabulated, and statistically analyzed via Microsoft Office Excel 2010 for Windows (Microsoft Cor., Redmond, WA, USA) and SPSS 22.0 for Windows (IBM Inc., Chicago, IL, USA). Continuous quantitative variables are expressed as the means ± SDs, medians (ranges) and 95% confidence intervals (CIs), and categorical qualitative variables are expressed as absolute frequencies (numbers) and relative frequencies (percentages). Continuous data were checked for normality via the Shapiro‒Wilk test. The paired t test was used to compare two dependent groups of normally distributed data, whereas the Wilcoxon signed-rank test was used for nonnormally distributed data. All tests were two-sided. A p value < 0.05 was considered statistically significant (S), a p value < 0.001 was considered highly statistically significant (HS), and a *p* value ≥ 0.05 was considered statistically insignificant (NS).

## Results

The mean age of the patients was 10.2 ± 1.39 years (ranging from 6 to 13 years). Among the patients, 8 were girls, and 12 were boys. Eleven patients had right-sided fractures, and nine had left-sided fractures. The mean follow-up period was 7 months (ranging from 4 to 9 months). There was one case of preoperative anterior interosseous nerve injury, which improved after 8 weeks, indicating neuropraxia. The demographic data indicated that 70% of the patients were either overweight or obese, 90% were either Gartland grade III or IV, and in 70% of the patients, high-energy trauma (such as sport participation and falls from height) was identified as the mechanism of injury (Table 1).

**Table 1:** Distribution of patients according to demographic data

Variable	*N* = 20	%
Sex Female Male	8 12	40 60
Side of lesion Left Right	9 11	45 55
Age (year) 6–8 years > 8–13 years Mean ± SD Median (Range) 95%CI	9 11 10.2 ± 1.39 8 (6–13) 7.43–10.76	45 55
Obesity Normal Overweight Obese	6 7 9	30 35 35
Time from injury to surgery (hrs) Mean ± SD Median (Range) 95%CI < 8 h > 8–48 h	19.15 ± 10.26 17 (8–48) 14.34–23.95 9 11	45 55
Fracture Gartland type IIb III IV	2 14 4	10 70 20
Mechanism of trauma High energy Low energy	14 6	70 30

Continuous variables were expressed as mean ± standard deviation (SD), median (Range) and 95%Confidence Interval of mean (CI), Categorical variables were expressed as number (No.) & percentage (%)

The time to union ranged from 4 to 6 weeks, with a mean of 5.15 ± 0.81 weeks. Union was assessed both clinically (no tenderness at the fracture site) and radiologically (by the presence of bridging callus in at least 3 of 4 cortices). In the present study, the mean operation time was 32.65 ± 4.38 min, and the average number of C-arm images used during the procedures was 25.3 ± 5.75. At the final follow-up, the radiological parameters for fracture reduction included the Baumann angle, the anterior humeral line, and the carrying angle. The mean Baumann angle was 70.05 ± 1.70 degrees [ranging from 67 to 73]. The anterior humeral line intersected the capitellum at the middle third in 16 patients and at the anterior third in 4 patients. The carrying angle ranged from 6 to 15 degrees, with a mean of 12.95 ± 3.85 degrees (Table 2). According to the Flynn clinical criteria [7], 15 patients achieved excellent outcomes (the total elbow range of motion loss and the carrying angle difference from the normal side were less than 5 degrees), whereas five patients were rated as having good outcomes (the total elbow range of motion loss and the carrying angle difference from the normal side ranged from 5 to 10 degrees). The mean total elbow range of motion was 135.15 ± 3.450.

**Table 2:** The final follow-up results of the current study

The final follow-up results		The studied patients (*N* = 20)	
		No.	%
*Time till union (weeks)*			
Mean ± SD		5.15 ± 0.81	
Median (Range)		5 (4–6)	
95%CI		4.76–5.53	
4 weeks		5	25
5 weeks		7	35
6 weeks		8	40
*Flynn criteria*			
Good		5	25
Excellent		15	75
*Operation time (min.)*			
Mean ± SD		32.65 ± 4.38	
Median (Range)		32.50 (25–40)	
95%CI		30.60–43.70	
*Baumann angle (0)*			
Mean ± SD		70.05 ± 1.70	
Median (Range)		70 (67–73)	
95%CI		70.85–69.26	
*Carrying angle (0)*			
Mean ± SD		12.95 ± 3.85	
Median (Range)		14 (1–16)	
95%CI		11.15–14.74	
*C-arm images (number)*			
Mean ± SD		25.3 ± 5.75	
Median (Range)		24 (13–31)	
95%CI		20.67–27.93	
*Total Elbow ROM (0)*			
Mean ± SD		135.15 ± 3.45	
Median (Range)		135 (128–140)	
95% CI		133.53–136.76	

Continuous variables are expressed as the means ± standard deviations (SDs), medians (ranges) and 95% confidence intervals (CIs). Categorical variables are expressed as numbers (numbers) and percentages (%)

We compared the Baumann angle, the carrying angle, and the total elbow range of motion of the fractured side to those of the normal contralateral side (Table 3). There was no statistically significant difference between the two sides for either the Baumann angle or the carrying angle. However, there was a statistically significant difference in the mean total elbow range of motion between the injured side (135.15 ± 3.450) and the contralateral normal side (138.55 ± 2.480). According to Morrey et al. [18], total loss of the elbow range of motion of up to 5 degrees compared with the normal side is functionally negligible, and total loss of up to 15 degrees has minimal functional limitations and is often asymptomatic.

Regarding complications, there were five cases of mild pin tract infection, all of which resolved within one week following the removal of the K-wires.

**Table 3:** Comparison of the final outcome results between the injured side and contralateral side

Measurements	Injured side (*N* = 20)	Contralateral side (*N* = 20)	Test	*p*-value (Sig.)
*Baumann angle (0)*				
Mean ± SD	70.05 ± 1.70	70.40 ± 3.18	– 0.022a	0.983
Median (Range)	70 (67–73)	70 (67–78)		(NS)
95%CI	70.85–69.26	68.91–71.90		
*Carrying angle (0)*				
Mean ± SD	12.95 ± 3.85	10.80 ± 2.28	– 2.606a	0.009
Median (Range)	14 (1–16)	11.50 (5–14)		(S)
95%CI	11.15–14.74	9.73–11.86		
T*otal Elbow ROM (0)*				
Mean ± SD	135.15 ± 3.45	138.55 ± 2.48	– 6.955b	< 0.001
Median (Range)	135 (128–140)	139 (135–143)		(HS)
95%CI	133.53–136.76	137.38–139.71		

Continuous variables were expressed as mean ± standard deviation (SD), median (Range) and 95%Confidence Interval of mean (CI); a: Wilcoxon Signed Ranks test; b: Paired t-test; *p*-value < 0.05 is significant; Sig.: Significance

## Discussion

Closed reduction and percutaneous pinning are the primary treatment for displaced ESCHFs. However, achieving an acceptable reduction via traditional closed reduction methods is not always feasible [19].

According to Sun and coworkers [20], the fracture type, mechanism of injury, and timing between injury and surgery are independent risk factors for failed closed reduction. Gartland type III fractures are particularly associated with a higher rate of open reduction. This is due to the complete loss of cortical contact, the absence of a periosteal hinge, and the potential for soft tissue entrapment. The mechanism of injury is another independent risk factor for failed closed reduction. High-energy trauma can lead to increased swelling of the elbow, marked fracture displacement, and comminution, complicating the reduction process. In the present study, we observed similar trends in our patient analysis. We found that 70% of the irreducible cases were classified as Gartland type III fractures, and 70% of the cases resulted from high-energy trauma.

The timing of surgery for SCHFs is still a controversial issue. While there is a general consensus that early closed reduction within the first 12–24 h is the best, the maximum delay for obtaining acceptable closed reduction is still controversial. Sun et al. [20] reported that the rate of open reduction increased from 10.2% to 29.1% if surgery was delayed beyond 8 h. Furthermore, if surgery was performed between 8 h and 5 days post-injury, the rate of open reduction increased to 65%. Skaggs & Flynn [10] emphasized that closed reduction should be attempted within 24 h and that after 72 h, open reduction is usually needed. Delayed presentation is usually associated with increased swelling at the fracture site, which obscures bony landmarks and makes fracture reduction more challenging. Heras et al. [21] recommended a delay of 5–7 days before performing reduction and fixation in cases of marked edema to allow soft-tissue recovery and avoid iatrogenic complications. Silva et al. [22] achieved near anatomical reduction in type IIb SCHFs treated 7 to 15 days after injury. From a pathological point of view, in the early reparative stage of fracture in children (3–7 days), the fracture ends become sticky, the mobility at the fracture site decreases, and closed reduction becomes difficult. The late reparative phase (7–14 days): The soft callus starts mineralizing into woven (hard) callus, so closed reduction after 10–15 days is difficult or even impossible [23]. In the present study, we did not observe any significant difference between patients who presented for treatment within 8 h of injury and those who presented after 8 h. Additionally, we excluded patients who had more than 10 days since the time of the injury.

Many studies have confirmed that obesity is an independent risk factor for failed SCHF reduction [24–26]. Obesity increases the risk of technical difficulties during the reduction and fixation of SCHF. Excess soft tissue can obscure anatomical landmarks and make manipulation, fluoroscopic visualization, and accurate pin placement more challenging. Many studies have reported an association between obesity and an increased degree of fracture displacement in patients with Gartlant type III fractures [25, 26]. We noted that 70% of our irreducible cases involved patients who were either overweight or obese.

Leitch and colleagues described a multidirectional unstable SCHF and classified it as a grade IV SCHF. This fracture is unstable in both flexion and extension due to the complete loss of the periosteal hinge both anteriorly and posteriorly. They recommended inserting two lateral K-wires in the unreduced position and rotating the image intensifier rather than the patient’s arm to achieve acceptable closed reduction [3]. We had four cases of irreducible type IV fractures, all of which were successfully reduced by the three-wire technique. With the aid of the distal wires, the distal fragment was easily manipulated and well controlled, helping with the fracture reduction and maintenance until final fixation. We also inserted the wires in an unreduced position and rotated the image intensifier rather than the patient’s arm.

Heffernan and colleagues reported a reversed oblique SCHF, where the fracture line in the sagittal plane extends from anterior and proximal to posterior and distal. This type of fracture is challenging to reduce via traditional closed methods because of the presence of an anterior spike in the distal fragment, which faces a posterior metaphyseal spike in the proximal fragment. They recommended maintaining the distal fragment in its displaced posterolateral position while translating it anteriorly to disimpact the proximal fragment [27]. In the present study, we included two reversed oblique ESCHFs. With the assistance of the three-wire technique, we were able to achieve posterior angulation of the distal fragment with the apex anterior and manipulate the fragments until they were offended, facilitating the fracture reduction.

Many studies have reported the use of posterior intrafocal K-wire for the correction of sagittal plane deformity. They used the posterior wire solely for reduction [28, 29] or for both reduction and fixation [30]. However, this technique is not recommended for fractures with posterior comminution or suspected neurovascular entrapment. Additionally, it is ineffective for correcting coronal or rotational deformities. However, with the three-wire technique, we were able to correct the fracture rotational deformity by rotating the proximal fragment internally or externally by the proximal joystick wire according to the fracture configuration. Additionally, the posterior comminution is not a contraindication for our technique as we do not depend on the posterior cortex for obtaining or maintaining the reduction. We depend on the wires for manipulating and controlling the fracture fragments.

Basaran et al. [31] utilized a proximal K-wire as a joystick to facilitate the correction of rotational deformities. They initially corrected the coronal plane deformity, then inserted a lateral K-wire for the fixation of the fracture fragments, and subsequently used the proximal joystick wire to address the rotational deformity. However, positioning of the proximal wire just distal to the deltoid insertion, which is far from the fracture site, results in less control over the proximal fragment. Additionally, if there is a residual sagittal plane deformity, it will be difficult to correct in the presence of the lateral K-wire.

Ma and coworkers described a technique using K-wires for the reconstruction of the medial and lateral column periosteal hinges in type IV SCHF. In their approach, two K-wires were inserted—one into the medial column and the other into the lateral column of the distal fragment—extending into the medullary canal of the proximal fragment. They concluded that this technique reduced the operation time, fluoroscopy exposure, and rate of incisions required [32].

Lin and coworkers described the use of a desharpened K-wire introduced at the lateral column of the distal fragment to enter the medullary cavity. They noted that as the K-wire advanced deeper into the medullary canal, it facilitated the correction of lateral coronal plane displacement. They concluded that the use of desharpened K-wires provided satisfactory reductions comparable to those of traditional methods, while also reducing the operation time and minimizing exposure to radiation from the image intensifier [33].

In the present study, we were able to manipulate the fracture fragments easily with the three wires. The three-wire technique facilitated the correction of the coronal, sagittal and rotational plane deformities. We did not have any patients who needed open reduction or reoperation. The mean operation time was 32.65 ± 4.38 min, which is comparable to the findings of Ma et al. [32] (32.32 ± 10.25 min) and Lin et al. [33] (32.88 ± 3.69 min). The average number of intraoperative fluoroscopies was 25.3 ± 5.75, which was slightly greater than that reported by Ma et al. [32] (15.24 ± 6.25) and Lin et al. [33] (20.62 ± 5.41). This increase may be attributed to the fact that our study focused exclusively on irreducible cases. Table 4 presents a comparison between our technique and other closed reduction techniques.

**Table 4:** Comparison between our technique and other closed reduction techniques

Study	Technique	Cases No	Mean age (years)	Operation time (minutes)	Fluoroscopy exposure	Clinical results	Radiological results	Complications
Ma et al. [32]	K-wire reconstruction of medial And lateral columns	25	7.12 ± 2.24	32.32 ± 10.25	15.24 6.25	23 of 25 are excellent and good. The mean total elbow ROM; 136.56 ± 6.49.	Mean Baumann angle; 73.10 ± 2.28. Shaft condylar angle; 36.84 ± 3.81.	Non mentioned.
Lin et al. [33]	Lateral intramedullary elastic reduction wire	50	8.5 ± 2.6	32.88 ± 3.69	20.62 ± 5.41	Flynn score; excellent and good rate was 98.00%	Baumann angle difference to normal side is 2.3 ± 0.6.	Non mentioned.
Lee and Kim [28]	Posterior pin leverage technique	21	-	68	-	All patients are either excellent or good.	Excellent and good without mentioning the angles.	2 cases of failed reduction, and open reduction was done.
Basaran et al. [31]	Proximal joy stick and lateral wire	13	7.8 ± 2.3	63.4 ± 26	-	7 excellent, 5 good and one fair.	Baumann angle difference to normal side is 4.3 ± 2.8. Carrying angle difference to normal side is 2 ± 2.1.	Non mentioned.
Kao et al. [34]	Posterior intrafocal pin for reduction and fixation	35	7.4 ± 2.2	-	-	The mean total elbow ROM 138.7 ± 9.4.	Baumann angle 70.9 ± 6.3. Anterior humeral line; Anterior third; 4, Middle third; 18, Posterior third; 12 Posteriorly lost; 1.	Non mentioned.
Our study	Three-wire technique	20	10.2 ± 1.39	32.65 ± 4.38	25.3 ± 5.75	Flynn; 15 excellent, 5 good. Mean total elbow ROM; 135.15 ± 3.45	Mean Baumann angle; 70.05 ± 1.70 Anterior humeral line; 16 middle, 4 anterior one third Mean carrying angle; 12.95 ± 3.85	5 cases of pin tract infection.

We had a single case of preoperative anterior interosseous nerve injury that presented with the inability to do the “OK” sign. We did gentle manipulation at the reduction stage and avoided multiple or forcible manipulations. Additionally, we started physical therapy after the back slab was removed, waiting for spontaneous nerve recovery. This case was resolved after 8 weeks, which is similar to that reported in the literature [35, 36]. Most of the preoperative anterior interosseous nerve injuries associated with ESCHFs are neuropraxia that resolves within 6–10 weeks [35, 36]. Additionally, the presence of preoperative anterior interosseous nerve injuries does not necessitate open reduction [37].

However, the current study has several limitations, including its small sample size, retrospective nature and relatively short follow-up period. Additionally, there is potential selection bias and a lack of blinding in the outcome assessment. Finally, we did not investigate the risk factors for failed closed reduction due to the absence of a control group.

## Conclusion

The three-wire technique is a straightforward and effective method for the closed reduction of irreducible ESCHFs. We propose a multicentric large prospective comparative study for displaced ESCHFs that compares the three-wire technique to the traditional Flynn reduction and fixation method.

## Data Availability

No datasets were generated or analysed during the current study.
